# Rasmussen’s encephalitis with persistent epilepsy in a young man

**DOI:** 10.1002/ccr3.3759

**Published:** 2021-01-09

**Authors:** Van Trung Hoang, Thanh Tam Thi Nguyen, Nhu Quynh Vo, Vichit Chansomphou, Cong Thao Trinh

**Affiliations:** ^1^ Department of Radiology Thien Hanh Hospital Buon Ma Thuot Vietnam; ^2^ Department of Radiology Hue University of Medicine and Pharmacy Hue Vietnam; ^3^ Department of Radiology Savannakhet Medical‐Diagnostic Center Kaysone Phomvihane Lao People's Democratic Republic; ^4^ Department of Radiology Hue Central Hospital Hue Vietnam

**Keywords:** chronic neurological disorder, hemimegalencephaly, persistent seizures, rasmussen's encephalitis

## Abstract

Rasmussen's encephalitis (RE) is an uncommon cause of the seizure. Important key findings of RE include intractable seizure activity in children, progressive atrophy of the involved hemisphere, and small hemisphere with the large ventricle.

## Graphical abstract

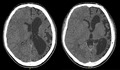

## 1 CASE DESCRIPTION

Rasmussen's encephalitis is a rare chronic neurological disorder, characterized by unilateral inflammation of the cerebral cortex, drug‐resistant epilepsy, progressive neurological, and cognitive impairment. We report a case of a 17‐year‐old male with persistent epilepsy, intellectual impairment, and cognitive deterioration.

**FIGURE 1:**
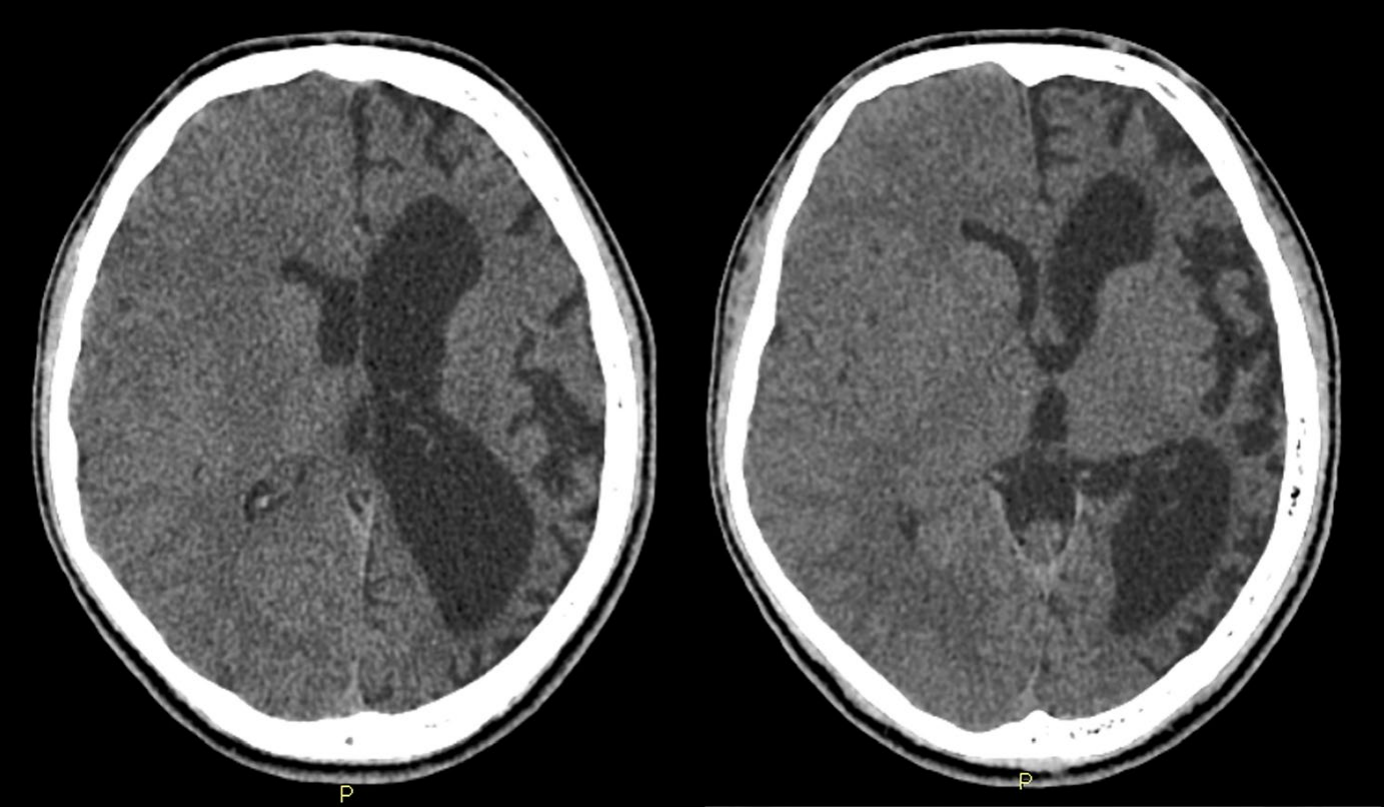
Noncontrast computed tomography of the brain shows atrophy of the left cerebral hemisphere with dilatation of the left lateral ventricle

A 17‐year‐old male was admitted to the hospital for persistent seizures that had gradually increased since 5 years ago. His right half was weakened gradually, and his memory was also deteriorating. He had been examined in many places but cannot diagnose the disease and only treat symptoms. This time at our hospital, his physical examination revealed intellectual and cognitive impairment. Computed tomography images showed atrophy of the left cerebral hemisphere (Figure 1). Clinical and imaging features were consistent with the diagnosis of RE. This patient had been attempted treatment with conventional therapies including steroids, plasmapheresis, and intravenous immunoglobulin. The condition had improved, but not completely.

RE is a rare chronic neurological disorder, characterized by unilateral inflammation of the cerebral cortex, drug‐resistant epilepsy, progressive neurological, and cognitive impairment. The etiology is unknown, but evidence supports an autoimmune basis.^1^ Usually, in the acute stage, there is a hyperintense signal on the T2‐weighted image that is often present in cortical or subcortical regions. The distribution is heterogeneous and temporal fluctuation. Thereafter, most patients present with unilateral enlargement of the ventricular system and hemisphere atrophy of the same side. RE requires a differential diagnosis from hemimegalencephaly. Treatment with immunotherapy can improve at an early stage, and at a later stage, hemispherectomy should be considered.^2^

## CONFLICT OF INTEREST

The authors declare no conflicts of interest.

## AUTHOR CONTRIBUTIONS

VTH: involved in patient management and wrote the manuscript. VTH, TTTN, NQV, and CTT: revised the manuscript. VTH and VC: reviewed the manuscript. All authors: read and approved the final manuscript.

## ETHICAL APPROVAL

Ethics approval was not required for this study.

## INFORMED CONSENT

Written informed consent was obtained for use of the clinical images.

## Data Availability

None.

## References

[1] Varadkar S , Bien CG , Kruse CA , et al. Rasmussen's encephalitis: clinical features, pathobiology, and treatment advances. Lancet Neurol. 2014;13(2):195‐205. 24457189 10.1016/S1474-4422(13)70260-6 PMC4005780

[2] Bien CG , Widman G , Urbach H , et al. The natural history of Rasmussen's encephalitis. Brain. 2002;125(Pt 8):1751‐1759. 12135966 10.1093/brain/awf176

